# Radiological Assessment of Bioengineered Bone in a Muscle Flap for the Reconstruction of Critical-Size Mandibular Defect

**DOI:** 10.1371/journal.pone.0107403

**Published:** 2014-09-16

**Authors:** Randa Al-Fotawei, Ashraf F. Ayoub, Neil Heath, Kurt B. Naudi, K. Elizabeth Tanner, Matthew J. Dalby, Jeremy McMahon

**Affiliations:** 1 University of Glasgow, Glasgow, United Kingdom; 2 Oral & Maxillofacial Radiology, Glasgow University Dental Hospital & School, Glasgow, United Kingdom; 3 Oral Surgery, Glasgow University Dental Hospital & School, Glasgow, United Kingdom; 4 Biomedical Materials, School of Engineering, University of Glasgow, Glasgow, United Kingdom; 5 Centre for Cell Engineering, University of Glasgow, Glasgow, United Kingdom; 6 Oral & Maxillofacial Surgery, Southern General Hospital, Glasgow, United Kingdom; University of Sheffield, United Kingdom

## Abstract

This study presents a comprehensive radiographic evaluation of bone regeneration within a pedicled muscle flap for the reconstruction of critical size mandibular defect. The surgical defect (20 mm×15 mm) was created in the mandible of ten experimental rabbits. The masseter muscle was adapted to fill the surgical defect, a combination of calcium sulphate/hydroxyapatite cement (CERAMENT™ |SPINE SUPPORT), BMP-7 and rabbit mesenchymal stromal cells (rMSCs) was injected inside the muscle tissue. Radiographic assessment was carried out on the day of surgery and at 4, 8, and 12 weeks postoperatively. At 12 weeks, the animals were sacrificed and cone beam computerized tomography (CBCT) scanning and micro-computed tomography (µ-CT) were carried out. Clinically, a clear layer of bone tissue was identified closely adherent to the border of the surgical defect. Sporadic radio-opaque areas within the surgical defect were detected radiographically. In comparison with the opposite non operated control side, the estimated quantitative scoring of the radio-opacity was 46.6% ±15, the mean volume of the radio-opaque areas was 63.4% ±20. Areas of a bone density higher than that of the mandibular bone (+35% ±25%) were detected at the borders of the surgical defect. The micro-CT analysis revealed thinner trabeculae of the regenerated bone with a more condensed trabecular pattern than the surrounding native bone. These findings suggest a rapid deposition rate of the mineralised tissue and an active remodelling process of the newly regenerated bone within the muscle flap. The novel surgical model of this study has potential clinical application; the assessment of bone regeneration using the presented radiolographic protocol is descriptive and comprehensive. The findings of this research confirm the remarkable potential of local muscle flaps as local bioreactors to induce bone formation for reconstruction of maxillofacial bony defects.

## Introduction

Loss of bone due to trauma, infection or resection of pathological lesions results in large, osseous, segmental defects of the facial skeleton which are difficult to reconstruct. Even in the best hands, inadequate vascularisation at the site of the bone defect (recipient site) has been the main obstacle for successful reconstruction with bone grafting [1]. Many strategies have been proposed for the management of mandibular surgical defects following bone loss, vascularized autogenous bone grafts are considered the most reliable method for reconstruction. However, this type of graft is not suitable if the patient has been subjected to radiotherapy or is suffering from peripheral vascular disease which compromises the blood supply to the surgical site. The harvesting of vascularised bone graft is associated with well documented morbidities [2]. Bone bioengineering using biomaterial, bioactive molecules and autogenous stem cells have been studied extensively and variable rates of success are reported [3]–[5]. Other studies have investigated various ways to induce angiogensesis and arteriogenesis which are essential for the bone regeneration process [6], [7]. The applications of vascular endothelial growth factors (VEGF), angiogenic proteins and hypoxia induced factor-1α to improve vascularity at the surgical site have also been reported [8]–[10].

More advanced surgical techniques were advocated to overcome the problems of the limited vascularity at the surgical defects “the recipient site” by utilizing local skeletal muscle flap to induce bone formation due to its reliable source of adequate blood supply [11]–[13]. The muscle has the propensity to induce bone formation because of its intrinsic osteogenic potential when exposed to osteogenic stimuli including bone matrix substitutes and bone morphgenic proteins (BMP) [14].

In clinical practice, plain radiographs are the most common method for the evaluation of bone regeneration [15]. However, this type of radiograph provides two diminsional (2D) representation of three diminsional (3D) structures, the superimposition of the medial and lateral surfaces confuses the analysis of bone regeneration. On the other hand the objective evaluation of radiographs and the application of a comprehensive scoring system are mandatory for the assessment of the quality and magnitude of the bioengineered bone. Radiographic assessment of bone regeneration should be comprehensive enough to record the dynamics of bone formation and the rate of degradation of bio-scaffold [16]–[19]. It has been recognized that cone beam computerized tomography (CBCT) is a reliable imaging modality for the 3D assessment of bioengineered bone [20]. The main advantage of this radiographic method is the reduction of radiation dose in comparison with conventional CT scanning [21].

Micro-computed tomography (µ-CT) is a sophisticated radiographic technique that permits quantitative morphometry of the bone structure in three dimensions [22]. The method produces high resolution images for accurate assessments of the microarchitecture of the bone tissue [23]. It allows the volumetric assessment of complex structures which consists of materials and tissues of variable densities which is the case of bone regeneration within radio-opaque biphasic calcium phosphate cement [24].

This study evaluated the reconstruction of critical size mandibular defects in rabbits using a pedicled masseter muscle flap which was injected with calcium sulphate/hydroxyapatite cement (CERAMENT™|SPINE SUPPORT), BMP-7 and rabbit mesenchymal stromal cells (rMSCs). Bone regeneration within the muscle tissue was monitored using plain extra-oral lateral oblique radiographs at three time points; 4, 8, 12 weeks post-operatively. CBCT, and Micro-CT assessments were carried out at 12 weeks following surgery. This article provides a comprehensive protocol for the assessment of bone regeneration process and highlights the importance of the objective 3D radiographic analysis and the assessment of the micro-structural details of the regenerated bone tissue.

## Materials and Methods

### Surgical procedure

**Figure 1:**
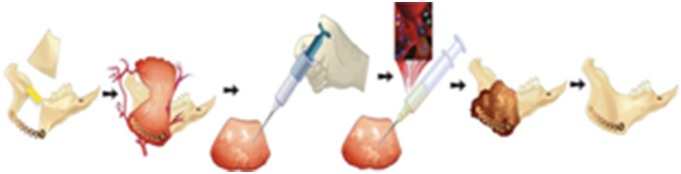
Diagrammatic illustation of the surgically created defect at the ramus of the mandible, fixation of the titanium plate, adaptation of the deep layer of masseter muscle into the defect followed by injection of Cerement™, BMP-7, and rMSc.

Approval was obtained from the Home Office of the United Kingdom under the Animals (Scientific Procedures) Act 1986 to carry out regulated procedures on living animals. Rabbit bone marrow aspiration, rMSCs isolation, culture, characterization, and assessment of cells to cement interactions were carried out by our team as reported previously [25]. The cement used in this study was injectable and commercially available (CERAMENT™ |SPINE SUPPORT, Bone Support AB, Sweden) which consisted of 60% calcium sulphate, 40% hydroxyapatite powder, and a liquid phase made of water and pacifier (iohexol). This study was carried out on ten New Zealand rabbits weighing 3.5–4.0 kg. The animals were obtained from a designated farm and kept in a dedicated animal house under veterinary supervision in the small animal research facilities. Each case was premedicated with 12.5 µg Fentanyl transdermal patch applied the afternoon prior to surgery. Fentanyl citrate & Fluanisone (Hypnorm, 0.5 ml/kg) was given intramuscularly 30 minutes before surgery and Midazolam (2 mg/kg) was administered intravenously at induction. The animals received endotracheal intubation and were given Insflurane, nitrous oxide, and oxygen. Oxygen saturation was monitored throughout the surgical procedure. A 2 cm right submandibular skin incision was performed to expose the angle of the mandible. This region was cut about 3–5 millimeters posterior to the last molar tooth, and the second cut was made 10 mm posterior to the first cut across the ascending part of the ramus of the mandible to create a surgical defect of 20 mm×15 mm. The upper border of the mandible was kept intact. To maintain the stability of the mandible a titanium plate was applied at the inferior border and secured in place by two screws, one on each side of the created defect. The deep layer of masseter muscle was dissected, reflected from its insertion and adapted into the created defect in the ramus of the mandible. The cement, 1.5 ml CERAMENT™|SPINE SUPPORT was mixed with 0.4 mg/ml BMP-7 (Op-1 Osigraft, Stryker Biotech, Ireland) and the mixture was injected into the masseter muscle flap. Once the material set, which took about 10 minutes, the prepared rMSCs suspension which contained three and half million cells was injected around the cement (Figure 1). This two injections protocol was based on our previous laboratory studies [25]. The surgical site was sutured in layers, the animals followed a standard postoperative protocol to minimize infection and to reduce postoperative pain. The surgical site was inspected and cleaned daily. Animals were kept on soft food for 3–4 days following surgery. Postoperative analgesic Meloxicam (NSAID) 0.2 mg/kg subcutaneous was given and prophylactic antibiotics cover using Oxytetracyclin 10% injection of 0.2 ml/kg. After full recovery, the rabbit was transferred to the normal holding cage and 10 ml saline was given S/C to avoid dehydration. The activity of the rabbit was monitored on a daily basis, as was the site of the operation for any bleeding or signs of infection. A mushy diet was offered to the animals, 0.16 ml Metacam (NSAID) was given S/C during the first 24 hours following surgery and on the 2nd post-operative day.

The rabbits were euthanatized three months following surgery by an overdose of IV sodium pentobarbitone (140 mg/kg). The mandibles were explanted and the surgical sites were examined. Samples were subjected to a comprehensive radiological evaluation as explained below. Histological assessments were carried out, the details are beyond the scope of this article.

### Plain radiographic assessment - oblique extra-oral film

Assessment during the lifetime of the animals was carried out by taking extr-oral lateral radiograph using dental occlusal films (Kodak Ultra-speed DF-50, Eastman Kodak Company, 3 NY, USA) on day 0 (day of operation) and at 4, 8, 12 weeks after surgery; the rabbits were sedated with IV Hypnorm 0.16 ml 30 minutes before taking the radiographs.

The rabbits were rolled and placed on their sides with the heads stretched backwards. The occlusal radiographs were placed against the surgical side of mandibular ramus. The X-ray tube was positioned 30 cm from and perpendicular to the radiographs. The tube was directed from the submandibular region of the opposite side. The X-ray machine was adjusted to 70 kV for 0.32 seconds and 10 mA. The quantity of bone formation was assessed using a validated scoring system [18], as shown in Table 2.1. Similar radiographes were taken for the contra-lateral control side.

**Table 1:** Grading system for the quality and quantity of radiographic bone regeneration.

Extent of bone formation	Description
Grade 0	No change compared to the immediate post-operative appearance regarding the size and the shape of the surgical defect
Grade 1	Traces of radio-dense materials in the surgical defect
Grade 2	Flocculent radio-density with flecks of calcification
Grade 3	Defect is bridged at least at one point with radio-dense material
Grade 4	Defect is bridged on both medial and lateral sides with material of a uniform radio-density, the ends of the cortices remains visible.
Grade 5	Same as Grade 3; at least one of the cortices is obscured by the new formed radio-opaque tissue.
Grade 6	Surgical defect is bridged by uniform radio-opaque tissue; which is completely indistinguishable from the surrounding bony edge.

**Table 2:** Scale used in calculating quantitative score (ΣQS) and its interpretation.

Grade	**∑**QS	Interpretation
0	0	0% radio-opacity
1–2	25%	25% radio-opacity
3–4	50%	50% radio-opacity
4–5	75%	75% radio-opacity
6	100%	100% radio-opacity

**Figure 2:**
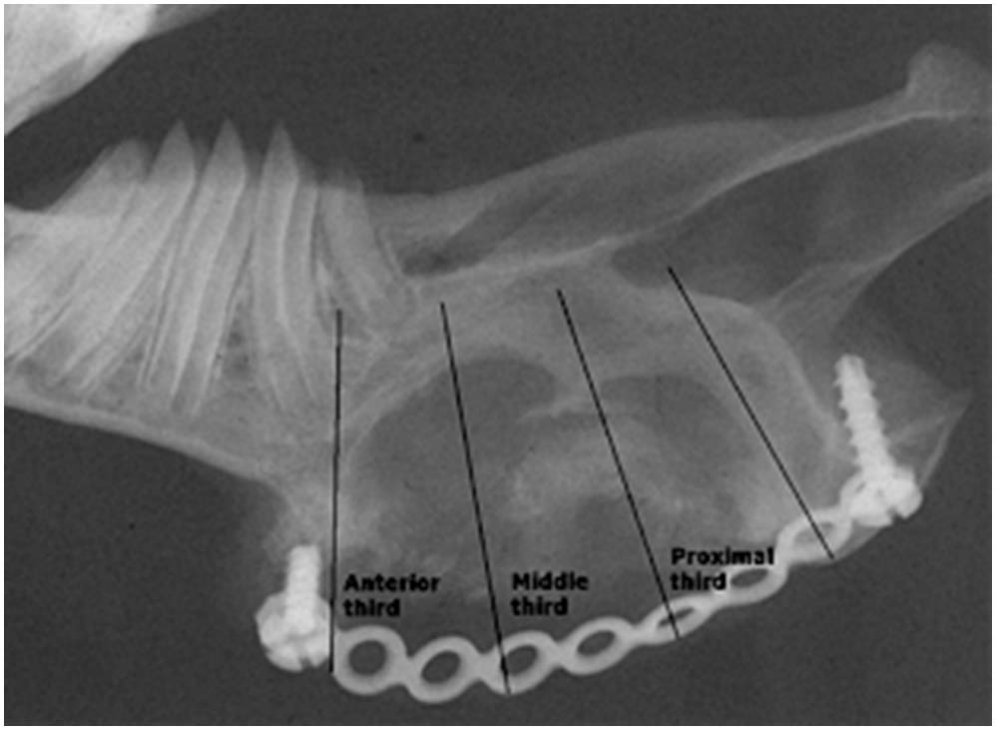
Radiographic image of the ramus of the mandible three months following surgery, showing the anatomical subdivision of the radiograph into anterior, middle, and posterior thirds for qualitative assessment.

To assess the pattern, radio-density, and quantity of the regenerated mineralised tissue (bone) in the created surgical defect, the radiographs were subdivided into thirds: anterior, middle, and posterior (Figure 2). The extent of bone formation was expressed in terms of the clinical qualitative score (CQS) and quantitative radiographic score (∑QS) which were developed by our research group [26]. This clinical qualitative score (CQS) represented the lowest score of each third of the examined region according to the described criteria in Table 1.

The quantitative scoring (∑QS) was calculated for the overall bone formation in each surgical defect. ∑QS is the average percentage of the three grades of bone formation in each surgical defect. This was calculated according to the following equation: ∑QS = {% GRADE anterior + %GRADE middle + %GRADE posterior}/3 (table 2).

### Cone beam computerised tomography (CBCT)

CBCT machine (i-CAT, Imaging Sciences International, Hatfield, UK) was adjusted at the following settings: 6 cm field of vision (FOV) scan, 120 kV at 20 sec, 0.3 mm pixel/voxel size, for radiographic image capture [27]. The explanted jaws were kept on a stable plastic mounting table, the fixation plate and the screws were left in place during image capture. Data was stored on optical discs to assess the cross-sectional of bone tissue and the volume of the regenerated bone.

**Figure 3:**
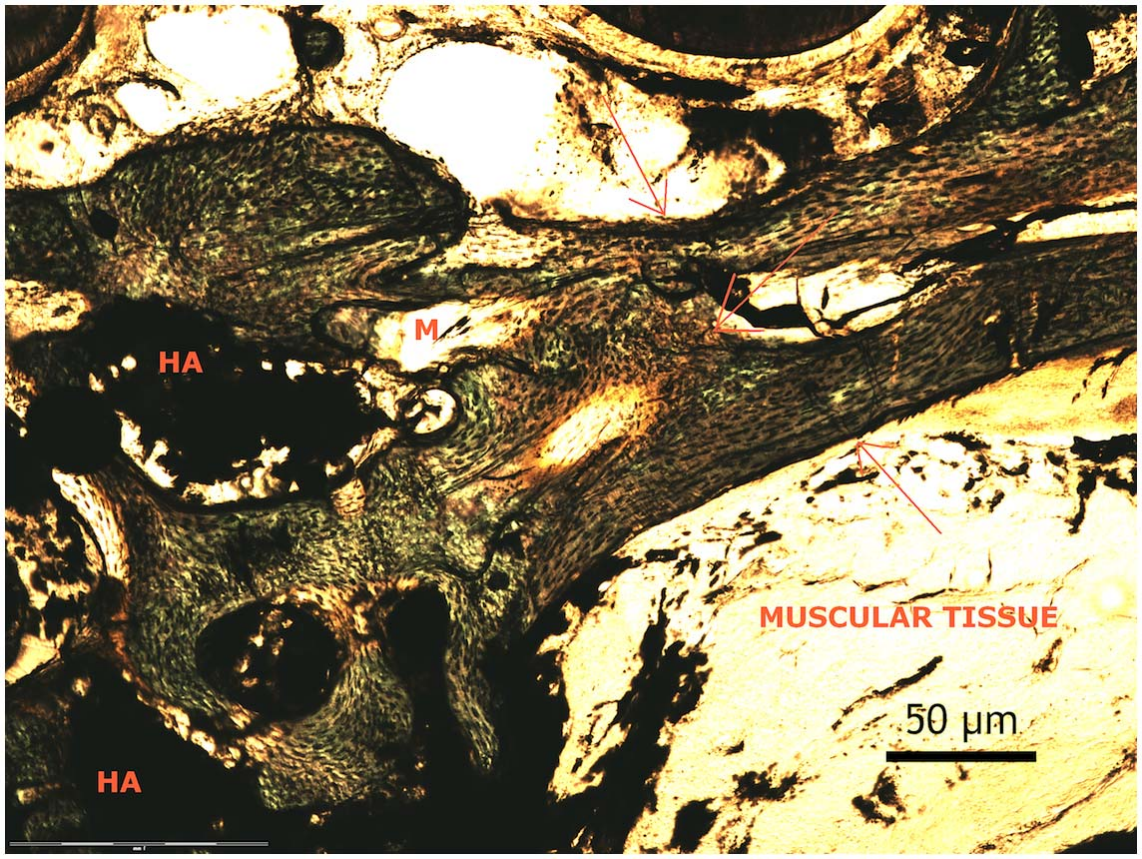
Histological picture of the regenerated tissue showing the pattern of newly formed bone (red arrows) within the muscle tissue (M) around the residual component of the cement (Hydroxyapatite) “HA”.

The histological assessment of the decalcified and undecalcified sections of the regenerated tissue confirmed bone formation within the muscle tissue (Figure 3), the details of the histology assessment are beyond the scope of this article.

### Assessment of bone volume and density

The volume of the regenerated bone was calculated from the coronal CBCT scans taken in contiguous 1.6 mm radiographic sections; a total of 10 coronal sections were retrieved and examined. The volume in cubic centimetres was calculated by multiplying the measured surface area of the radio-opaque mass of each section by 1.6 “the thickness of each section” and by the number of examined sections. The mean value and the standard deviation “SD” of all the specimens were calculated for statistical analysis. Means and SD of the bone mineral density were also estimated from the radiographic grayscale value of the Hounsfield unites “HU” [28].

### Micro-CT analysis

**Figure 4:**
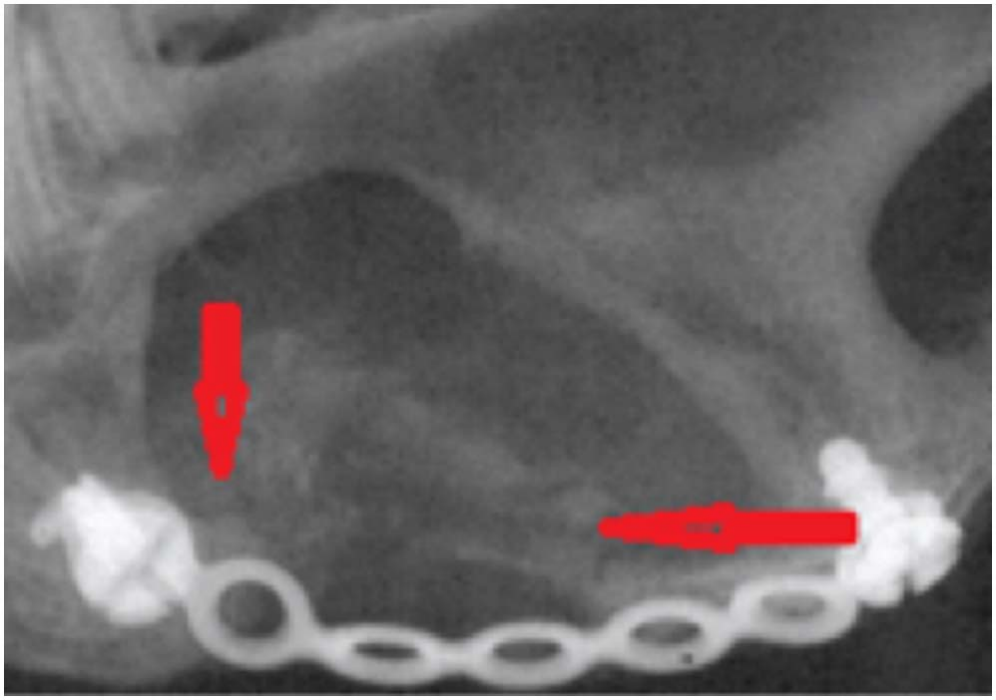
Micro CT scanning for one of the samples showing the Field of Vision (FOV) that was selected for all the samples; blue arrows indicate the boundaries of FOV, the anterior arrow (A) refers to area of the last premolar tooth; the last proximal screw represents the posterior limit (P) of the FOV, vertically the FOV extended 1 cm above the ramus notch denoted with yellow arrow.

**Figure 5:**
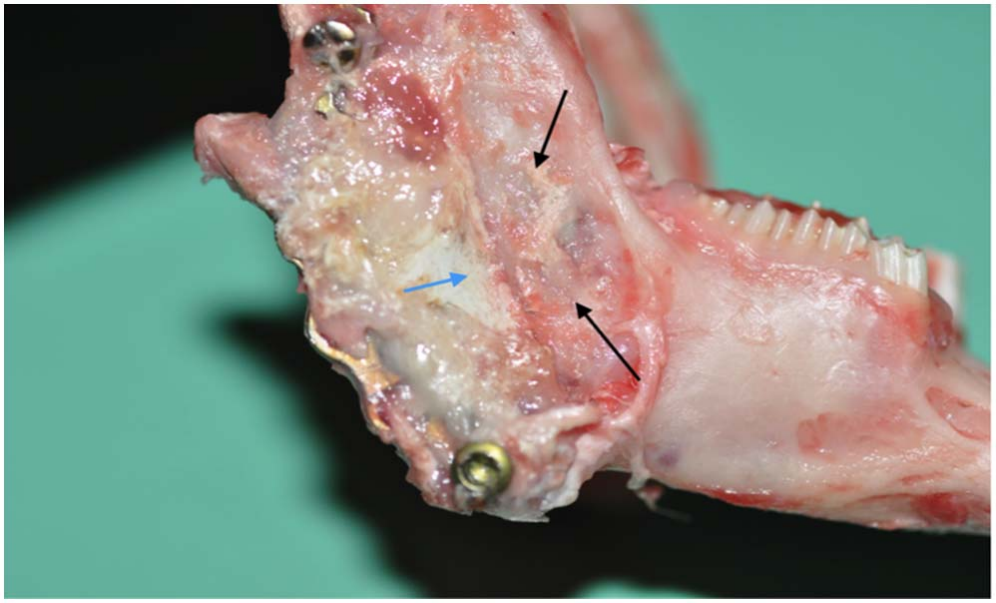
Clinical picture for the operated side of a harvested mandible, showing the muscle tissue occupying most of the area of the created defect. The figure shows the fixation screw and the titanium plate (red arrows) across the inferior border of the defect. The blue arrows denote an area of residual cement, and areas of calcified tissue at the superior border of the defect (black arrows).

This was carried out using a micro-CT system (Skyscan 1172 HR; Skyscan, Aartselaar, Belgium). One non-operated hemi-mandible was scanned as a control. The following micro-CT settings were applied: X-ray energy levels at 100 kV, electric current at 167 µA, and integration time of 120 ms with 17 µm voxel size, and 360° rotation. The captured and reconstructed images were segmented using a threshold algorithm [29]. Thresholding was applied to separate the regenerated bone or the remaining cement from the surrounding soft tissue, this process was based on subjective matching of images followed by objective global thresholding which was optimized for the used cement “biphasic calcium phosphate cement” [30] (Figure 4). To determine the field of view (FOV) from the sagittal micro-CT images the following reference points were placed; anterior point marked 5 mm posterior to the last molar, and a posterior point at the angle of the mandible tangent with the ramus notch (Figure 5). Three-dimensional analysis was performed, bone volume (BV) was measured, bone volume fraction was calculated as the ratio of BV to the total volume (TV), and the remaining cement volume (CV) was calculated using Image J software (version 10.3) [6]. This was achieved by calibrating the scale bar of the software, changing the image into gray scale at 8 bit, followed by selection of the field of view. The image was processed in a binary format; the areas of interest were measured by counting the number of pixels/µm of the selected region. The interface between the newly generated radio-opaque calcified tissue and the surrounding bone was examined. Trabecular thickness (TbTh) (or pore wall) and trabecular separation (TbSp) (or pore diameter), trabecular number (TbN), degree of anisotropy and bone mineral densities were calculated by direct measurements.

## Results

The rabbits completed the course of the study without significant adverse effects. Clinically, a thin layer of hard tissue regenerate appeared between the muscle tissues that emerged from the most inferior side of the graft. Fibrous tissue was found around the titanium plate. Bone overgrowth was observed at the area around the proximal (posterior) screw in most of the cases. An area of white rubbery mass was observed between muscle fibers which was the un-degraded remaining of the injected cement (Figure 5).

### Plain radiographic assessment “oblique extra-oral film”

**Figure 6:**
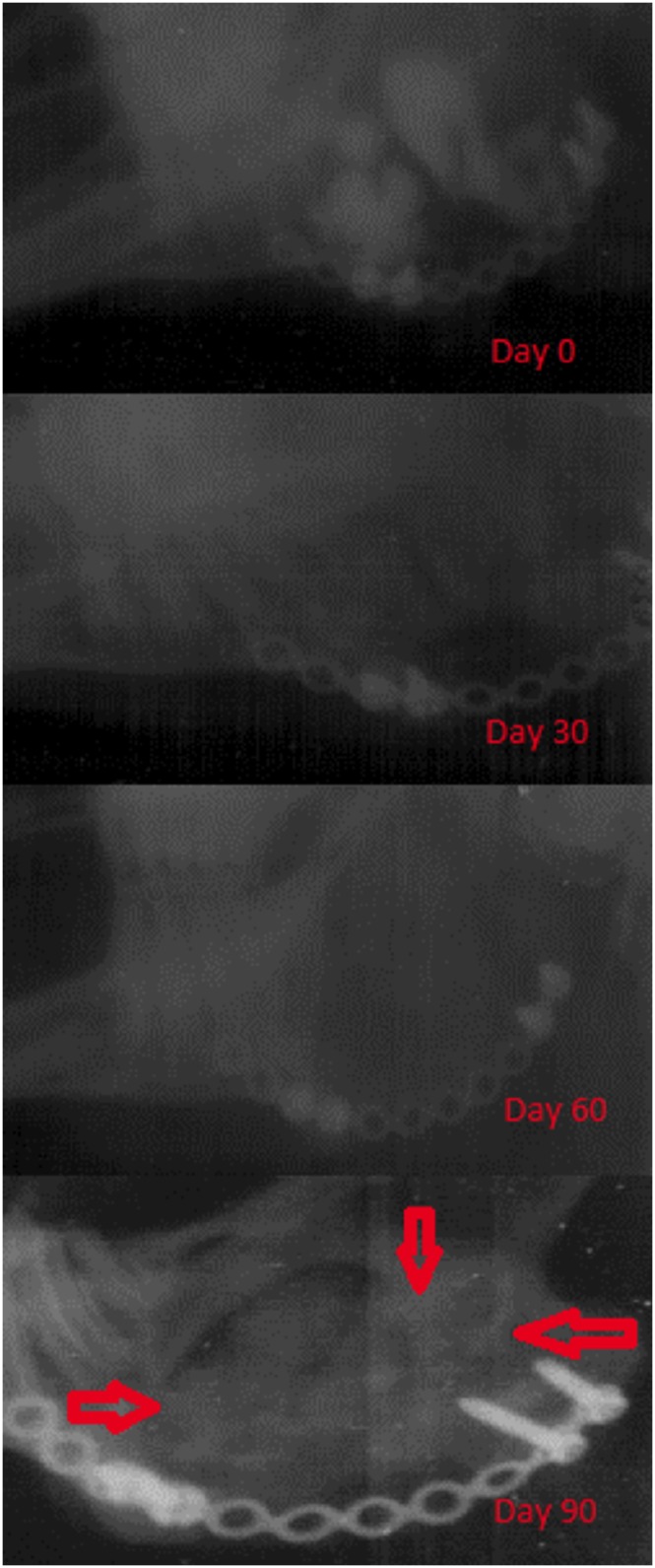
Radiographic images taken at different time points. A) immediately after surgery which shows the injected cement as radio- opaque patches, B) One month after surgery showing the disappearance of radio-opaque patches of cement, C) Two months following surgery, the radiograph shows homogeneous radio-opaque shadow, D) Three months postoperative radiograph showing radio-opaque mass occupying the area of the created defect.

At day 0 (immediately following surgery) the injected cement appeared as bright radio-opaque patches covering the surgical defect. Radiographs taken at 1 month post-surgery showed complete disappearance of the bright radio-opaque patches (Grade 1). This was due to the rapid absorption of the iohexol, the pacifier of the liquid phase of the cement. This image was considered the baseline for radiographic analysis. The radiographs taken 2 months following surgery showed sporadic radio-opaque areas in close approximation with the defect’s borders attempting to bridge the gap of the surgical defect (Grade 2). Areas of mixed radio-opacity and radiolucency were present at the centre of the defect. At 3 months post-surgery, the centre of the defect was occupied with a heterogeneous radio-opaque masses (Grade 3) extending from the centre of the defect. A more homogenous radio-dense mass appeared at the proximal end of the surgical defect (Figure 6). In one case complete bridging of the surgical defect with the radio-dense structure was noted (Grade 4). Table 3 summarizes the estimated quantitative scoring of the regenerated tissue for all the examined case. The mean percentage of radio-opaque tissue present in the created defect was 46.6%±15%.

**Table 3:** Shows the estimated quantitative scoring of different anatomical sections of the defect taken from the radiographs, ∑QS represents the percentage of the regenerated tissue for all the radiographs that were analyzed for each rabbit (Rb).

Rabbit no.	Radiographic examined sections grading and the total quantitative scoring using average percentage			
	Proximal	Middle	Anterior	Total ∑QS
Rb1 (151)	5	3	5	66.6±8.4
Rb2 (152)	5	4	3	58.3±12.7
Rb3 (214)	3	1	0	25±20
Rb (215)	2	1	1	25±19
Rb5 (218)	4	3	4	66.6±8.9
Rb (217)	4	2	2	41.6±13.8
Rb (216)	5	4	5	66.6±7.9
Rb (239)	4	2	5	50±20
Rb (240)	0	0	4	25±20.9
Rb (241)	1	3	4	41.6±17.6

The mean ∑QS reading was 46.6% (25–66.6).

### CBCT for volumetric bone assessment & evaluation of bone density

**Figure 7:**
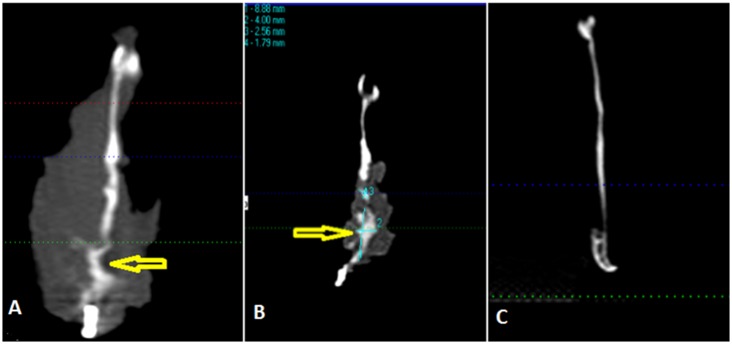
Coronal radiographic scan through the ramus of the mandible; the thickness of the slice is 1.6 mm. A, shows the bone regeneration area (yellow arrow). B, shows linear measurement of the newly formed bone. C shows coronal section of the bone of the contra-lateral side.

CBCT assessment and evaluation of 3D radiographic image revealed the presence of notable calcified tissue of a heterogeneous nature inside the masseter muscle at the site of the created defect (Figure 7). Areas of hypo-calcification were readily detectable within the muscle tissue. More cases showed calcified radio-opaque areas at the proximal third of the defect with no clear demarcation between the regenerated tissue and the surrounding native bone. The assessment confirmed that the volume of the radio-opaque tissue within the muscle flap was more than of the original bone at the region of the defect (ROD). In 4 cases there was an increase in volume of calcified tissue by 35%±15% compared to the volume of the ROD. However, this has to be interpreted carefully due to the fact that large areas of radio-opacities were detected around the periphery rather than in the centre of the defect. In the other six rabbits the mean estimated volume of regenerated bone was 302 mm^3^±120 mm^3^ which constituted 63.85%±20% of the volume of ROD (Table 4). The radio-opacity of the thickened bone at the margin of the defect was 35%±25% and was denser than the contra-lateral non-operated control side.

**Table 4:** Data from CBCT analysis, showing the estimated volume of radio-opaque tissues present at the defect & the mineralized density at the centre and the borders of the defect for all the rabbits (Rb).

Rabbit no.	CBCT examination				
	Average volumeof the radio-densetissue/mm^3^	Percentage ofradio-dense massin relation to ROD/%	HU of theRegeneratedtissue	HU at thecontrol side	% of border tissuedensity in relationto the host bone/%
Rb1 (151)	871±13	160±11	748±285	761±250	+130
Rb2 (152)	2365±9	70±15	528±127	706±345	+25
Rb3 (214)	181±13	41±13	576±165	1003±242	+23
Rb (215)	315±19	67±5	666±160	931±233	+70
Rb5 (218)	196±20	39±8	918±234	824±268.8	+42
Rb (217)	456±27	120±14	1056±223	968±285	+75
Rb (216)	486±23	98±10	1470±234	836±300	−4
Rb (239)	656±17	122±6.7	1412±267	990±325	+9
Rb (240)	686±10	139±6	1390±241	1153±281.6	+45
Rb (241)	400±22	68±5	1294±253	1066±321	+38

### Micro-CT analysis

**Figure 8:**
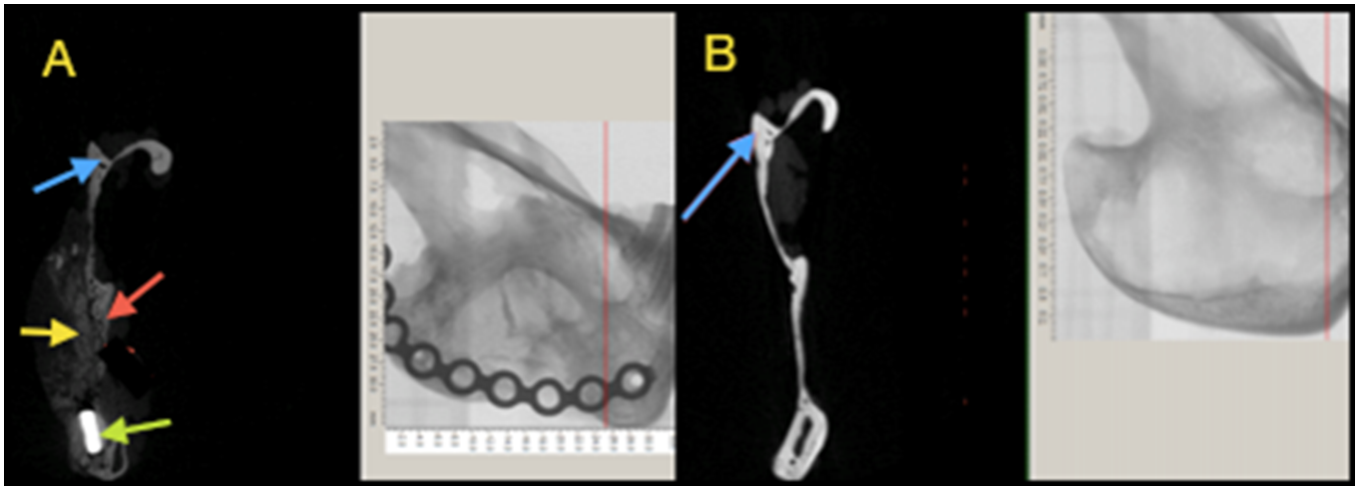
Micro-CT screen of the coronal section at the anterior third of the defect showing an area of tissue regeneration. A, shows an area of bone bridging the surgical defect, red arrows is pointed medially toward the native bone, the yellow arrow refers to the radio-opacity of the newly generated tissue that is indistinguishable from the native bone, the green arrow is marking the titanium plate. B shows the corresponding anatomical region of the contra lateral non-operated side.

**Figure 9:**
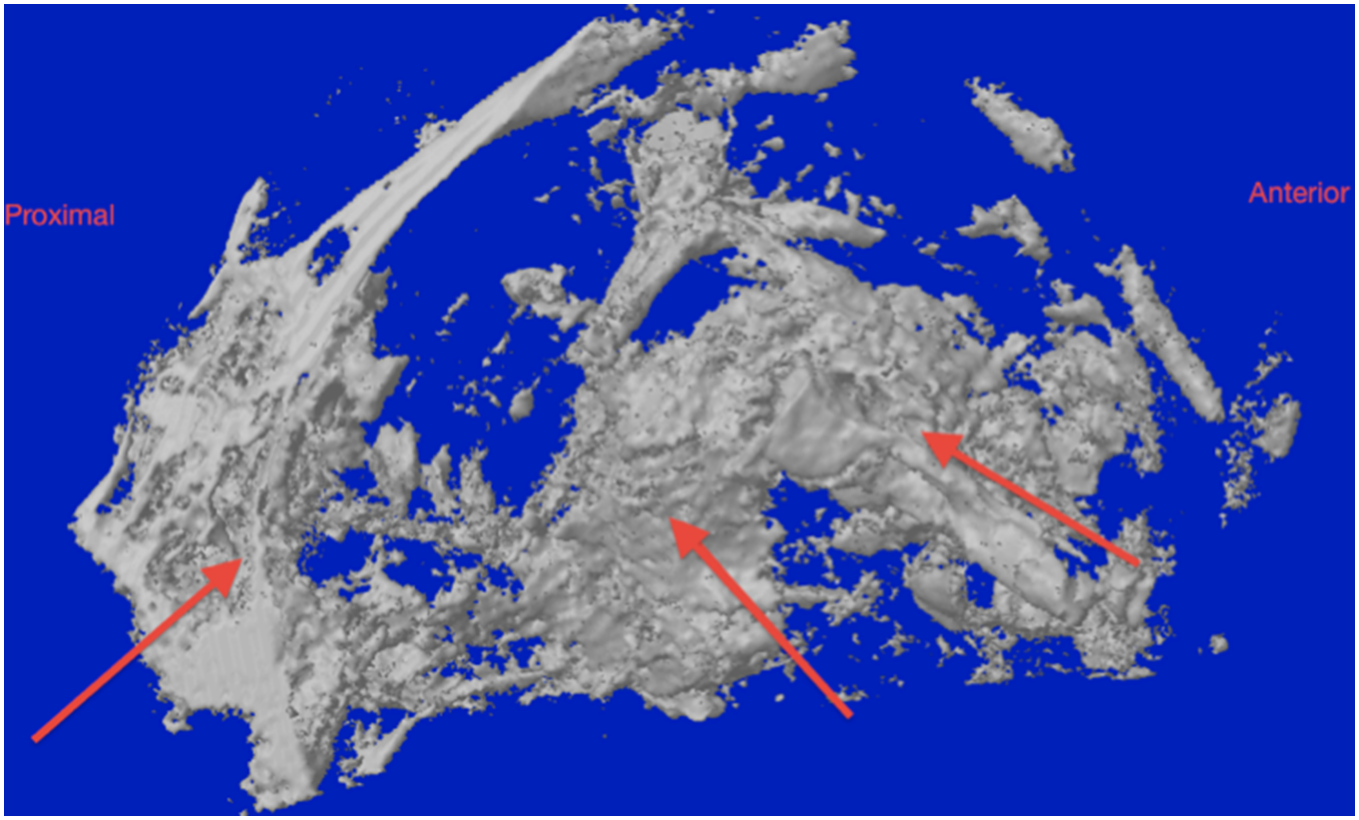
Sagittal section of the 3D model of one of the cases showing the surgical defect filled with a radio-dense mass which appears irregular in shape (red arrows).

The boundary between the regenerated tissue and the surrounding native bone was indistinguishable (Figure 8). The regenerated bone tissue at the defect site was either medial or lateral to the surrounding native bone. The surface morphology of the regenerated tissue was rough in comparison with the surrounding bone which is suggestive of an active remodeling process. The presence of hydroxyapatite crystal remnants of the injected cement may have contributed to the apparent surface roughness (Figure 9).

**Figure 10:**
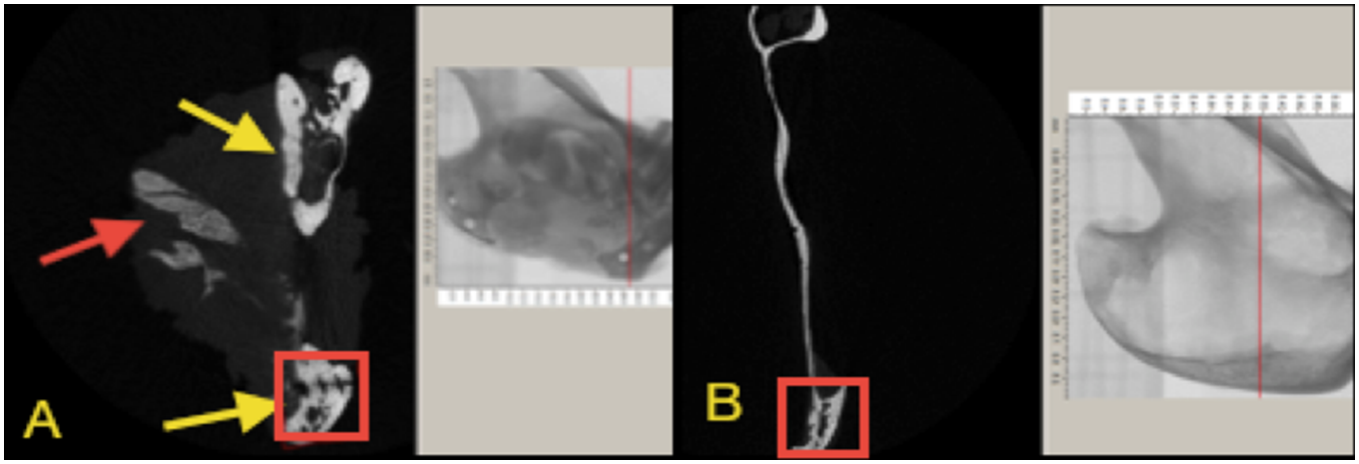
Micro-CT screen image of a coronal slice through the area of the defect showing bone bridging the gap (red box). A: Red arrow represents area of bone regeneration located lateral to the defect, the red box represents areas of bone overgrowth (yellow arrows) at the interface between the newly generated bone and the native bone. B: The contra-lateral non-operated side of the mandible, the red box represents the corresponding region as seen in figure A (Field of view = 10.0×7.0×4.0).

**Figure 11:**
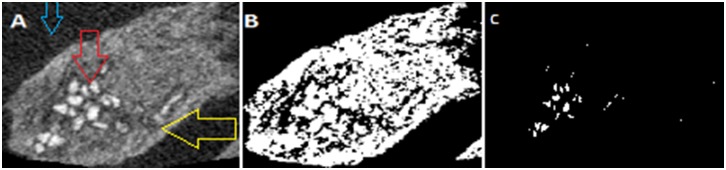
Axial view of micro-CT indicates an area of bone formation which appears gray (yellow arrow), the surrounding soft tissue appears black (blue arrow) and area of the remaining cement in white color (red arrow). B: Binary picture of the threshold of the same image. C: The white particles of the remaining cement analyzed by Sky scan image analysis software.

The coronal cross-sections confirmed that the regenerated tissue has a trabecular pattern and were thicker medio-laterally than the contra-lateral non-operated side (Figure 10). The average bone volume fraction (BV/TV) of the regenerated bone within the ROD was 36.2%±14%. However, the mean volume of the new bone formed outside the ROD was 237.8 mm^3^±50.9 mm^3^ which was 5 times the volume of the bone within the ROD, and the percentage of the remaining cement was 15.9% ±10% (Figure 11). At the area of interface between the regenerated and native surrounding bone, the mean bone volume fraction was 28.8 mm^3^ which was double the volume of bone on the same representative anatomical region at the non-operated side (Table 5).

**Table 5:** Summary of the micro-CT quantitative analysis (mean percentages±SD) and trabecular characteristics of the newly regenerated tissue in comparison with the bone of control sides at three months following surgery (n = 3).

*Structural index*		*Rb 151*	*Rb152*	*Rb 216*	*Rb 239*	*Control*
BV mm^3^ (inside ROD)		0.60±0.20	0.48±0.14	0.44±0.05	0.37±0.03	0.99±1.40
BV mm^3^ (outside ROD)		84.9±13.4	26.9±7.0	320.7±23.0	249.1±29.9	
CV		8.6±0.6	7.6±2 3	0.07±0.06	0	-
% BV/TV in ROD		1.45±5.9	1.17±4.8	0.16±.02	0.16±0.08	1.00±0.58
% BV/TV at area of interface		1.2±0.4	1.78±1.39	1.8±0.20	1.29±7.0	1.00±0.07
Bone Surface area (BS)/Total surface area (BS/TS)*		0.51	0.52	0.99	0.59	1.00
	Tb Th (mm)	0.21±0.04	0.42±0.06	0.48±0.20	0.34±0.10	0.56±0.14
	Tb N (mm^−1^)	1.21±0.9	0.75±0.20	0.57±0.02	0.87±0.22	0.32±0.20
Trabecular analysis	Tb Sp (mm)	0.7±0.1	1.19±0.5	1±0.2	0.9±0.2	1.4±0.1
	DA	1.63±0.90	1.72±0.34	1.78±0.33	1.79±0.4	2.24±0.51
	Bone density g/cm^2^	0.59±0.20	0.58±0.05	0.97±0.24	0.56±0.07	0.99±0.09

*Image analysis using ImageJ (ImageJ, 1.46 h, Wayne Rasband, National Institutes of Health, USA).

BV = Bone Volume, CV = Cement Volume, ROD = Region of the defect, BV/TV = Bone Volume fraction, TV = Tissue volume, BS = Bone surface area, BS/TS = Bone surface area fraction, TbTh = Trabecular thickness, Tb N = Trabecular number.

Tb Sp = Trabecular separation, DA = degree of anisotropy.

**Figure 12:**
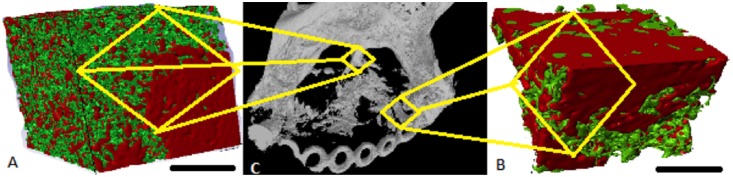
Micro-computed tomographic images. A illustrates bone generation at the superior margin of the surgical defect as shown in C. B is for the regenerated bone at the anterior border of the defect as shown in C. The red areas represent a mixture of a radio-dense tissue of bone and cement, the green areas represent the muscle tissue (marker bars = 1 mm).

**Figure 13:**
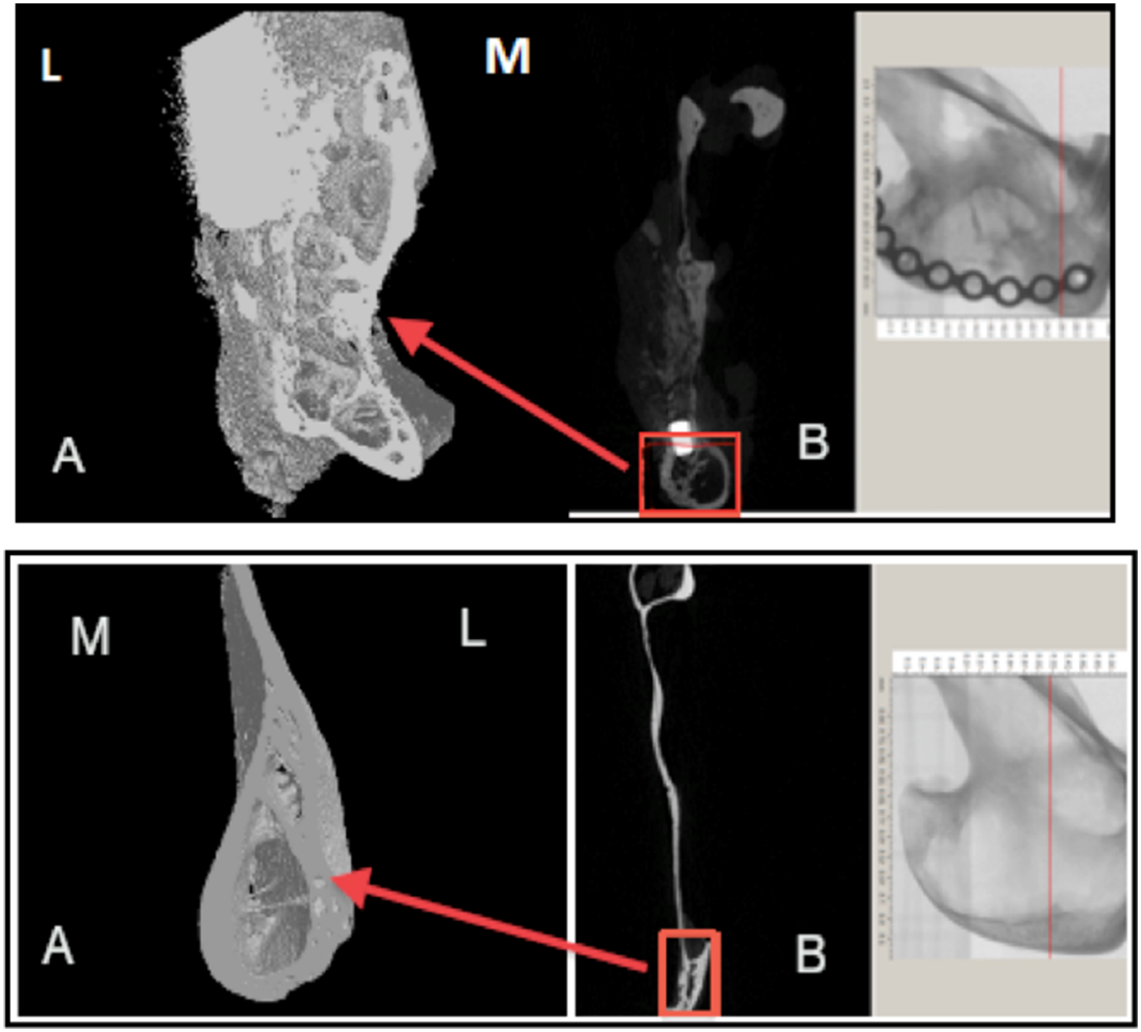
**a:** 3D model of the micro CT scan of an area of bone regeneration at the anterior region of the surgical defect demonstrating the enlargement of trabecular bone in the medio-lateral direction (M-L), B shows the coronal section through the same area (red box) of the regenerated bone. **b:** The corresponding 3D micro-CT scan of the same region of the contra-lateral non-operated side.

With regard to bone architecture for the same regions, the average TbN/mm^3^ of the newly regenerated bone was double that of the contra-lateral non-operated side. TbTh, and TbSp were 63.3%±4.6%, and 73%±7.6% respectively in comparison with the contra-lateral (Figure 12). The degree of anisotropy (DA) was close to the contra-lateral side, and bone density was 85% ±50 of that of the contra-lateral non-operating side (Figure 13a, 13b).

The newly formed bone attained more volume than the ROD; the bone also showed a thinner but more condensed trabecular pattern with a lower degree of anisotropy which suggests that the bone had a fast deposition rate and had undergone an active remodeling process.

**Figure 14:**
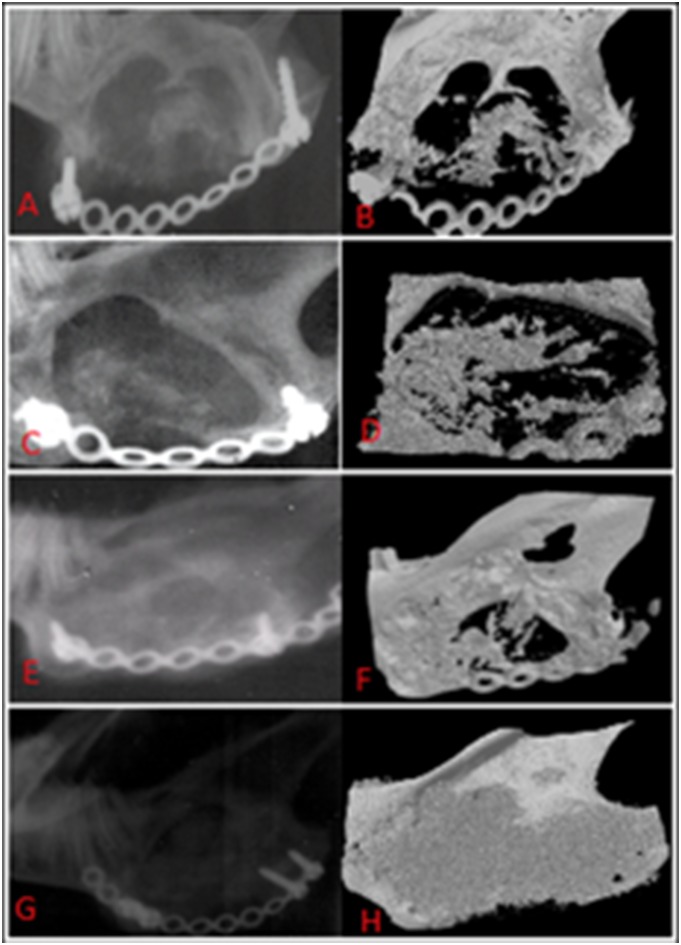
A comparison between the plain radiographs (A, C, E, G) and the corresponding micro-CT images (B, D, F, H). Analysis on the micro-CT scans using image analysis software revealed that 65.4%±14% of the ROD was occupied by calcified tissues which was consistent with plain radiographic quantitative assessment of bone formation.

On the other hand, 2D analysis of the sagittal micro CT images using image analysis software (Image J, version 10.1) revealed that 65.4%±14% of the ROD was occupied by calcified regenerated tissues. The latter was almost consistent with plain radiographic quantitative assessment of bone formation with a mean score of 60.4%±9% (Figure 14).

More bone formation was observed at the borders and lateral to ROD. Figure 12 shows a schematic representation of the pattern of bone regeneration at the border, the centre, medial side and at the lateral side of the ROD.

## Discussion

This study demonstrates the remarkable potential for the use of muscle flap, injectable cement, BMP-7 and rMSC to induce bone formation. The muscle flap provided the required blood supply for bone bioengineering and acted as a bioreactor for bone formation. On average, more bone formation was detected within the muscle flap in comparison to the contra-lateral side, but the defect was not fully reconstructed with the regenerated bone due to the difficulty of injecting the cement at the centre of the defect.

The most widely available diagnostic tool for bone assessment is plain X-ray; despite its shortcomings it allowed assessment of bone regeneration during the life of the animal in this study. Cook’s scoring system [18] combined with the quantitative scoring system ∑QS provided a comprehensive measurement of the quantity of bone formation at the surgical bone defect, which was consistent with the morphometeric analysis of the micro-CT images. In this experimental model the application of a radio-dense material, the ceramic cement, which had a similar radio-opacity to that of bone, added to the complexity of studying the pattern of bone regeneration.

CBCT provided more precise information about the actual bone formation in comparison with 2D radiographs [31], [32]. In this study, the radio-opaque mass of the regenerated tissue had a large bucco-lingual dimension as shown in the coronal section. The volume of regenerated bone at the ROD was on average 35%±15% in comparison to the bone of the contra-lateral non-operated side. This finding was due to the fact that the injection of the cement included the entire mass of the muscle flap within the defect. Micro-CT was an essential tool to investigate the nature of the interface between the regenerated calcified tissue and the surrounding native bone and to explore the trabecular pattern of the regenerated bone [33], [24]. Micro-CT facilitated the differentiation between the structure of the remaining cement and the regenerated calcified tissue, this was achieved by adjusting the thresholding of the radiographic images manually. The global thresholding for biophasic calcium phosphate cement has been documented [24]. The estimated average new bone was 36.2% inside the region of the defect in all assessed cases. The mean volume of the newly formed bone outside the ROD was 237.8 mm^3^±50.9 mm^3^ which was 5 times the bone volume inside the ROD. The magnitude of the remaining cement was variable among the examined samples, the average percentage was 15.9% ±1%0. This was expected as the resorption rate of calcium sulphate component of CERAMENT™ |SPINE SUPPORT is faster than the hydroxyapatite part which takes longer to dissolve; a similar finding was reported when the cement was implanted in the abdominal muscle of rats [34]. The thin trabecular pattern and the increased number of the trabeculae of the newly formed bone suggest that it had a fast deposition rate and active remodelling process [35]. The observed lower degree of anistropy of the newly formed bone compared to the non-operated side is most probably due to the reduced extra cellular mineral deposition of the regenerated tissue [36]. The reduced anisotropy is due to the presence of HA crystals attached to the bony trabeculae.

This study demonstrated the value of the comprehensive radiographic assessment using various imaging modalities and highlighted the importance of various scoring systems to quantify bone formation for better understanding of the pattern of calcified tissue regeneration. It provides the first comprehensive radiology based 3D investigation to assess bone regeneration within muscle flaps in the oro-facial region. The findings confirm the ability of injectable cement loaded with BMP-7 and seeded with MSCs to stimulate bone regeneration and restore the trabecular bone structure within three months of injection inside a local muscle flap in the maxillofacial region. Nevertheless, complete conversion of the muscle into bone and full reconstruction of the defect was not achieved. This was partially due to the physical nature of the injected cement that prevented the diffusion of the material at a micro level between the fibres of the muscle tissue. The muscle flap obliterated the surgical defect making it clinically difficult to insure that the injected cement had uniformly reached the desirable location within the ROD. This may have been achieved with a thinner pedicled muscle that could be fully enclosed within the ROD. On the other hand one could argue that use of full thickness muscle flap is essential to maintain the integrity of the blood supply which is essential for bone regeneration. It has been reported that muscle volume and surface area are detrimental factors that govern the amount of bone regeneration [14].

## Conclusions

This study provided the proof of the concept that bone formation could be induced within a pedicled muscle flap in the maxillofacial region using an injectable cement loaded with BMP and seeded with rMSCs which has clear potential clinical applications. Further development and refinements of this approach are required before the clinical use in maxillofacial bone reconstruction.
